# Assessment of Glycosylated Hemoglobin Outcomes Following an Enhanced Medication Therapy Management Service via Telehealth

**DOI:** 10.3390/ijerph18126560

**Published:** 2021-06-18

**Authors:** Jennifer M. Bingham, Jennifer Stanislaw, Terri Warholak, Nicole Scovis, David R. Axon, Jacques Turgeon, Srujitha Marupuru

**Affiliations:** 1 Tabula Rasa HealthCare, Tucson, AZ 85701, USA; nscovis@trhc.com; 2 College of Pharmacy, University of Arizona, Tucson, AZ 85721, USA; jstanislaw@pharmacy.arizona.edu (J.S.); warholak@pharmacy.arizona.edu (T.W.); axon@pharmacy.arizona.edu (D.R.A.); marupuru@pharmacy.arizona.edu (S.M.); 3 Tabula Rasa HealthCare, Precision Pharmacotherapy Research and Development Institute, Orlando, FL 32827, USA; jturgeon@trhc.com; 4 Faculty of Pharmacy, Université de Montréal, Montreal, QC H3C 3J7, Canada

**Keywords:** type 2 diabetes, pharmacist, glycosylated hemoglobin, medication therapy management, comprehensive medication review, telehealth, T2DM

## Abstract

(1) Background: Regular contact with a medication therapy management (MTM) pharmacist is shown to improve patients’ understanding of their condition; however, continued demonstration of the value of a pharmacist delivered comprehensive medication review (CMR) using enhanced MTM services via telehealth is needed. The study aimed to describe a pilot program designed to improve type 2 diabetes mellitus (T2DM) management through enhanced condition specific MTM services. (2) Methods: This retrospective study included patients with T2DM aged 40–75 years who received a pharmacist-delivered CMR between January and December 2018. An evaluation of glycosylated hemoglobin (HbA1c) values 3 months pre- and post-CMR was performed. Wilcoxon signed-rank and chi-square tests were used. (3) Results: Of 444 eligible patients, a majority were female (58%) with a median age of 70 years. Median HbA1c values post-CMR were lower than pre-CMR (median 7.1% range 4.5–13.6; median 7.4% range 4.5–13.9, respectively; *p* = 0.009). There were fewer participants with HbA1c >9% post-CMR (*n* = 66) than pre-CMR (*n* = 80; *p* < 0.001) and more with HbA1C <6.5% post-CMR (*n* = 151) than pre-CMR (*n* = 130; *p* < 0.001). (4) Conclusion: This program evaluation highlighted the value of an enhanced condition specific MTM service via telehealth. Patients had improved HbA1c values three months after receiving a single pharmacist delivered CMR.

## 1. Introduction

Medication therapy management (MTM) services aim to optimize medication use, reduce medication-related problems, and reduce overall healthcare costs. One component of MTM services is an annual comprehensive medication review (CMR), which may be conducted by pharmacists or other qualified providers in a variety of ways, including in-person at provider offices and community pharmacies, or via the telephone [1,2]. It is known that MTM pharmacists play an integral role in assessing the patient’s understanding of his/her conditions and therapeutic regimens through regular contact and accessibility [3]. Yet, patients do not typically receive regular follow-up contact from MTM pharmacists beyond the annual CMR encounter.

Evidence linking pharmacist involvement to improved clinical outcomes and patient empowerment has led to the expansion of the pharmacist’s role in chronic disease management for several chronic conditions [4,5,6,7,8,9]. An example of this is in the management of type 2 diabetes [10]. For example, one study found that, for patients with type 2 diabetes, pharmacist delivered care significantly improved their ability to self-manage their condition, as seen by decreased glycosylated hemoglobin (HbA1c) clinical values 6 months post intervention [11]. Research also shows that through MTM counseling on lifestyle modifications (e.g., physical activity, nutrition), disease state management, and medication adherence, pharmacists make an important contribution to the diabetes care team [12]. Hence, MTM services are a crucial component of ensuring optimal outcomes in diabetes care given their ability to improve medication safety and effectiveness.

Although previous work has demonstrated the economic, clinical, and humanistic benefits of pharmacist delivered MTM services [8,9,10,11], an enhanced, condition specific MTM program for patients with type 2 diabetes mellitus (T2DM) has not yet been evaluated in the literature. A pilot, condition specific, pharmacist delivered MTM program was therefore implemented by a national MTM provider to empower self-management of tT2DM, optimize medication regimens, address gaps in care continuity, improve glycemic control, reduce risks of diabetes-related complications, and improve chronic condition management for patients with T2DM. Patients were identified by their respective health plan based on eligibility criteria set forth by the Centers for Medicare and Medicaid Services and then contacted by the national MTM provider. The purpose of this paper is to describe, within the context of MTM, a pilot program designed to improve T2DM management through enhanced condition specific MTM services integrated into an annual pharmacist delivered CMR service.

## 2. Materials and Methods

### 2.1. Description of Program

The CMR included an interactive, systematic assessment of patient-specific health information and medications to identify and resolve drug therapy problems and to improve health outcomes. The CMR was conducted using an audio-only telehealth (i.e., telephone call) application. Quality checks were performed on CMRs conducted by the pharmacists to ensure consistency and standardization.

First, the medication list was reconciled within the software program (RxCompanion™, Tucson, AZ, USA) and medication names, strengths, doses, routes, frequencies, and indications were recorded and assessed for drug-drug interactions, medication safety concerns, and appropriateness using telehealth. Next, patient allergies and reactions were recorded and assessed for contraindications. Pharmacists at the call centers were assisted by proprietary software that raised alerts for medication non-adherence, therapeutic duplications, and missing guideline-directed therapy.

Next, the pharmacist conducted a condition specific review via telephone. If the patient was diagnosed with T2DM, pharmacists were expected to address each of the following through teach-back education: (1) fasting blood sugars; (2) patient understanding of how to identify and manage hypoglycemia and hyperglycemia; (3) patient knowledge of how their most recent HbA1c compared to the previous value; (4) whether their HbA1C was uncontrolled based on provider recommendations; and (5) a review of results with self-monitoring of blood glucose. The pharmacist used these findings to have meaningful conversations about lifestyle factors to improve blood glucose management and address specific times of the day that were problematic for the patient.

Based on the patient response, the pharmacist provided teach-back education and an individualized written summary to empower self-management of T2DM. The patient was mailed a personalized medication list in the Centers for Medicare & Medicaid Services standardized CMR format upon completion of the CMR. The pharmacist directly contacted the provider via telephone or facsimile if they identified patient issues related to access to medication, therapeutic recommendations warranting a dosage change. The pharmacist referred the patient to their provider for further diabetes management and/or suggested a diabetes educator or dietician as needed.

### 2.2. Description of Sites

The MTM service provider offered an annual standardized CMR to qualified patients based on benefits eligibility. The MTM service provider started providing services via telehealth in 2006. Five national MTM call centers employed by the MTM service provider were included in the pilot program and presented a suite of MTM services to meet the performance needs of health plans and patients, mainly through pharmacist-delivered telehealth medication reviews. The centers consisted of a team dedicated to improving health, wellness, and chronic disease management through MTM services adopting an interprofessional team model that included: pharmacists, pharmacy technicians, student pharmacists, pharmacy residents, nursing students, and registered nurses.

### 2.3. Study Population

Patients were included in this program evaluation if they received an annual CMR in 2018 from a pharmacist employed by the MTM service provider. Additional patient criteria included: age 40 to 75 years; diagnosis of T2DM denoted by international classification of disease (ICD)-10 coding (e.g., E11.0–E11.9); and, had a presence of one or more oral antihyperglycemic prescriptions based on claims data. The Centers for Medicare & Medicaid Services (CMS) assess certain oral antihyperglycemics in performance metrics to analyze medication adherence for diabetes medications, which include: biguanides, sulfonylureas, thiazolidinediones, dipeptidyl peptidase-4 (DPP) inhibitors, incretin mimetics, meglitinides, or sodium-glucose co-transporter-2 (SGLT2) inhibitors. Medicare beneficiaries who only use insulin are not included in this CMS metric, and were subsequently excluded from the study. Additionally, patients who were 40 years of age or younger and older than 75 years were excluded from the study.

### 2.4. Data Collection and Analysis

This program evaluation used retrospective patient data from one MTM service provider between 1 January 2018 and 31 December 2018. The variables of interest included: (1) age; (2) gender; (3) race/ethnicity; (4) number of medications; (5) HbA1c three months pre-CMR; and (6) HbA1c three months post-CMR. Health marker data was provided by health plan to the national MTM provider. A pre-post study design was used. For nominal data, a chi-square test was used. For internal level data with a non-normal distribution, a Wilcoxon signed-rank test was used. Descriptive data were also calculated. All tests used an a-priori α level of 0.05. Data analysis was conducted using IBM SPSS Statistics v2015 (IBM Corp, Armonk, NY, USA). This program evaluation was approved by the institutional review board (No. 1911128095).

## 3. Results

A total of 444 patients were included in the program evaluation, of which 264 were female and 180 male. The median age of patients was 70 years of age (range 40–75). The median number of medications per patient was 21 (range 9–60). Additional baseline patient demographic information is outlined in Table 1.

Median HbA1c values post-CMR were lower than pre-CMR scores (median 7.1% range 4.5–13.6 and median 7.4% range 4.5–13.9, respectively; Z = −2.60, *p* = 0.009). An HbA1c threshold of greater than 9% was used to identify patients at highest risk for microvascular and macrovascular complications, as well as mortality. There were fewer participants with HbA1c >9% post-CMR (*n* = 66) compared to pre-CMR (*n* = 80; *p* < 0.001), and there were more participants with HbA1C <6.5% post-CMR (*n* = 151) compared to pre-CMR (*n* = 130; *p* < 0.001). A subgroup analysis was conducted to assess the impact in adults and the elderly. Of 444 patients, 104 (23%) were adults and 340 (75%) were older adults. Additional data concerning the number and percent of patients in each HbA1c strata pre- and post-CMR are in featured in Table 2.

## 4. Discussion

Our study highlights the effect of an enhanced condition specific MTM service designed to improve HbA1C control in patients with T2DM via telehealth. The most important finding was the significant difference between median HbA1c values pre- and post- CMR. This provides preliminary evidence to support the value of an enhanced diabetes specific MTM service in preventing the risk of microvascular and macrovascular complications associated with an HbA1c >9% [13]. The evidence also supports the value in assisting patients to achieve a desired HbA1c <6.5%, especially those who have a longer remaining life expectancy, fewer comorbidities, and a lower risk of hypoglycemia. Hence, we demonstrated in this study that the telehealth approach, by itself, had a significant impact on HbA1C control and represents another complimentary option to the use of medical devices, biosensors, or smart phone applications to support patients in their own homes.

MTM services are a crucial component of ensuring optimal outcomes in diabetes care [14]. The MTM pharmacist plays an integral role in assessing the patient’s understanding of his/her condition and therapeutic regimens through regular contact and accessibility [3]. One study found that, for patients with T2DM, pharmacist-delivered information significantly decreased HbA1c values and improved patient knowledge [11]. Another study found improved indicators following pharmacist provided MTM services [15]. Anderson et al. also found improved T2DM health markers following telehealth MTM pharmacist services [8]. In addition to managing T2DM, MTM service provider pharmacists also help reduce cardiovascular related complications and morbidity. Research shows that through MTM counseling on lifestyle modifications, disease state management, and medication adherence, pharmacists make an important contribution to the diabetes care team [12].

It is well known that preventative care is one of the most efficient ways to maintain patient health and to reduce healthcare costs [16]. Moczygemba et al. found that telephonic MTM programs reduced both medication- and health-related problems [10]. Thus, CMRs via telehealth can be thought of as preventative healthcare. The results of this study suggest that pharmacists can provide preventative care that may help to address unmet patient needs stemming from primary care provider shortages in the United States.

Telephonic CMRs are an effective method to enhance patient care and to reduce healthcare expenditures because they can optimize medication therapy and empower patients [16]. Another benefit of telephonic CMRs is that they can provide access to pharmacists who speak the patient’s preferred language, thus increasing culturally responsive patient care [17]. This is applicable because the MTM service provider in our study provides CMRs via telehealth to patients in over 30 languages. An additional advantage of telephonic CMRs is improved ease for patients to reconcile medication lists while remaining in their residences, rather than transporting pill bottles to another site and unintentionally forgetting one at home [1]. Future research should evaluate the relative association of each of these components on patient outcomes.

The results of our pilot program further suggest a role for MTM pharmacists in providing preventative care that may help to address unmet patient needs stemming from primary care provider shortages in the United States. One novel aspect of this program was the use of a telephonic condition specific review designed to assist patients with poorly controlled T2DM. The condition review allowed the pharmacist to discover patient specific challenges in glucose management and offered customized solutions based on their HbA1c and fasting blood glucose goals. In addition, the condition reviews were an effective method to enhance patient care by optimizing medication therapy and empowering patients. These results support the need for pharmacists to fill gaps in care subsequent to national provider shortages in the United States of America [16].

The successes of the pilot program provides lessons learned to other MTM providers aiming to improve quality measures for patients with T2DM. These findings parallel other studies that have shown pharmacist-delivered medication counseling and healthcare education to be valuable in improving clinical values [17].

### Limitations

This pilot evaluation study design prohibited the establishment of a causal relationship between the CMR and diabetes outcomes, as it did not control for length of the CMR, class of antihyperglycemic medication, specific changes to the medication list, patient-specific responses to the condition review, other comorbidities, or whether the patient sought services from their provider during the three-month time post-CMR. Hence, the study was not able to capture the clinical significance, nor the cost-effectiveness of the outcomes observed as there were limitations in data available to the research team. The study also did not capture longitudinal effects on clinical outcomes beyond three months post-CMR. In addition, the large proportion of patients with unknown race and ethnicity limits generalizability. Thus, these pilot program findings are not generalizable to all Medicare beneficiaries. In future studies, it would be prudent to collate data from the telehealth application regarding the length of the consultation. It is also suggested that researchers collect specifics from the software program on the type of medication and compare differences in the list pre- and post-intervention. One final suggestion is to integrate a complimentary follow-up consultation with the patient to assess for diabetes management from other healthcare providers.

## 5. Conclusions

This program evaluation highlights the value of pharmacist delivered CMR telehealth services aimed to decrease HbA1C values in patients with T2DM. Future research should evaluate the impact in a more diverse population in other countries over a longer time to determine the cost effectiveness and clinical significance of the intervention.

## Figures and Tables

**Table 1:** Baseline characteristics for participants (*n* = 444).

Characteristic	Median (Range)
	N (%)
Gender Male Female	180 (40) 264 (58)
Race/Ethnicity Asian Black White Unknown/Other Hispanic	8 (2) 32 (7) 37 (8) 337 (76) 30 (7)
Note: Percentages may not equal 100% due to rounding and missing values.	

**Table 2:** Comparison of HbA1c values pre-comprehensive medication review (CMR) intervention and post-CMR intervention.

	Pre-CMR	Post-CMR	*p*-Value
Total population *N* = 444			
HbA1c	N (%)	*N* (%)	
<6.5%	130 (29)	151 (34)	<0.001
6.5–9.0%	234 (52)	227 (51)	<0.001
>9.0%	80 (18)	66 (15)	<0.001
HbA1c *	Median (range)	Median (range)	*p*-value
	7.4 (4.5–13.9)	7.1 (4.5–13.6)	0.009
Adults (40–64 years of age) *N* = 104			
HbA1c	N (%)	*N* (%)	
<6.5%	24 (23)	40 (38)	<0.001
6.5–9.0%	51 (49)	46 (44)	<0.001
>9.0%	29 (28)	18 (17)	<0.001
HbA1c *	Median (range)	Median (range)	*p*-value
	7.8 (4.8–13.2)	7.1 (4.5–12.1)	0.002
Elderly (65–75 years of age) *N* = 340			
HbA1c	N (%)	*N* (%)	
<6.5%	106 (31)	111 (33)	<0.001
6.5–9.0%	183 (54)	181 (53)	<0.001
>9.0%	51 (15)	48 (14)	<0.001
HbA1c *	Median (range)	Median (range)	*p*-value
	7.2 (4.5–13.9)	7.4 (5.0–13.6)	0.256

HbA1c: glycosylated hemoglobin; * HbA1c data had a skewed distribution, hence median and range were reported. Differences between the pre- and post-groups were calculated using the Wilcoxon signed-rank test given that the data were dependent (i.e., the same people were included in the pre- and post-groups).

## Data Availability

The data are not publicly available due to proprietary concerns.
